# Emotional Intelligence and Well-being in Adolescents: a Systematic Review and Meta-analysis

**DOI:** 10.1192/j.eurpsy.2022.1770

**Published:** 2022-09-01

**Authors:** D. Llamas-Díaz, R. Cabello, A. Megías-Robles, P. Fernández-Berrocal

**Affiliations:** 1 Faculty of Psychology. University of Málaga, Department Of Basic Psychology, Málaga, Spain; 2 Faculty of Psychology. University of Granada, Department Of Developmental And Educational Psychology, Granada, Spain

**Keywords:** Subjective Well-Being, meta-analysis, Adolescents, Emotional Intelligence

## Abstract

**Introduction:**

Adolescent´s subjective well-being (SWB) can be improved through the training of emotional intelligence (EI).

**Objectives:**

The goal of this study is to determine the general link between EI and SWB in adolescents, to analyze the affective (AWB) and cognitive components (CWB) of SWB, and to investigate the moderating effect of EI models on both types of SWB.

**Methods:**

We searched PsycINFO and WOS from inception to December 2020. Eligible studies reported an association between EI and SWB in adolescents aged from 10 to 19 years using instruments that directly measure SWB. Two meta-analyses were conducted, one for the relationship between EI and AWB and the other for EI and CWB.

**Results:**

A total of 41 studies were included, of which 37 were pooled in the meta-analyses. We obtained a significant positive relationship between EI and AWB (estimated effect size = 0.35) and between EI and CWB (0.29). Concerning EI models, self-report ability showed an estimated effect size of 0.33 for AWB and 0.28 for CWB. For the self-report mixed model, we found an estimated effect size of 0.42 for AWB and 0.38 for CWB.

**Conclusions:**

Establishing a quantitative relationship between SWB and EI makes it possible to implement both clinical and educational prevention measures. Introducing EI training in educational and clinical settings can increase SWB, which could significantly impact the prevention of emotional disorders in adolescents.

**Disclosure:**

No significant relationships.

